# Dose–response relationship of sleep apnea therapy and healthcare use in patients with comorbidities

**DOI:** 10.1093/sleep/zsaf333

**Published:** 2025-10-27

**Authors:** Atul Malhotra, Suyog More, Naomi Alpert, Jean-Louis Pépin, Kate V Cole, Caleb Woodford, Adam V Benjafield, Peter A Cistulli, Kimberly L Sterling, medXcloud Group, Atul Malhotra, Jean-Louis Pépin, Peter A Cistulli, Kimberly L Sterling, Carlos M Nunez

**Affiliations:** Department of Medicine, University of California San Diego, La Jolla, CA, United States; Resmed Science Centre, Halifax, Nova Scotia, Canada; Resmed Science Center, San Diego, CA, United States; HP2 Laboratory (Hypoxia: Pathophysiology), Grenoble Alpes University, Grenoble, France; Resmed Science Center, San Diego, CA, United States; Resmed Science Centre, Halifax, Nova Scotia, Canada; Resmed Science Center, Sydney, Australia; Charles Perkins Centre and Faculty of Medicine and Health, University of Sydney, Sydney, Australia; Resmed Science Center, San Diego, CA, United States

**Keywords:** obstructive sleep apnea, positive airway pressure, adherence, healthcare resource use, real-world evidence

## Abstract

**Study Objectives:**

Obstructive sleep apnea (OSA) has complex interactive relationships with several other conditions. Previous research suggests that consistent adherence to positive airway pressure (PAP) therapy can reduce healthcare resource utilization (HCRU) in comorbid populations. We hypothesized that PAP therapy use would be associated with dose-dependent improvements in HCRU among patients with OSA and comorbid chronic obstructive pulmonary disease (COPD), type 2 diabetes, depression, heart failure, or atrial fibrillation.

**Methods:**

We analyzed a linked dataset of medical/pharmacy claims data and objective PAP usage data for adults with newly diagnosed OSA between January 2015 and May 2021. Comorbidities were defined by at least two healthcare encounters or at least one hospitalization with the relevant diagnosis in the year before PAP initiation (index). HCRU outcomes included all-cause hospitalizations and emergency room (ER) visits over 12 and 24 months post-index.

**Results:**

Among 377 830 patients with OSA (mean age: 51.7 years; 57.7% male), 6.6% had COPD, 18.7% type 2 diabetes, 16.5% depression, 4.2% heart failure, and 5.2% atrial fibrillation. Across all comorbidity cohorts, PAP usage was associated with a dose-dependent reduction in HCRU over 12 and 24 months. Risk-adjusted analyses showed HCRU benefits beginning at 2 to less than 4 hours of average nightly PAP use. Each additional hour of use was associated with a 4.1%–6.2% reduction in hospitalizations and ER visits (all analyses *p* < .0001).

**Conclusions:**

PAP therapy use is associated with dose-dependent reductions in HCRU among patients with OSA and major comorbidities. These findings may support data-driven reimbursement policies and highlight the value of treating OSA in complex patient populations.

## Teaser

**Statement of Significance** With data from 377 830 patients, increasing PAP usage was associated with a 4.1%–6.2% reduction in HCRU per additional hour of PAP use. A clear dose-dependent relationship between PAP usage and HCRU was found over both 12 and 24 months. These results support the maximization of PAP usage in patients with major comorbid conditions.

## Graphical Abstract

**Graphical Abstract:**
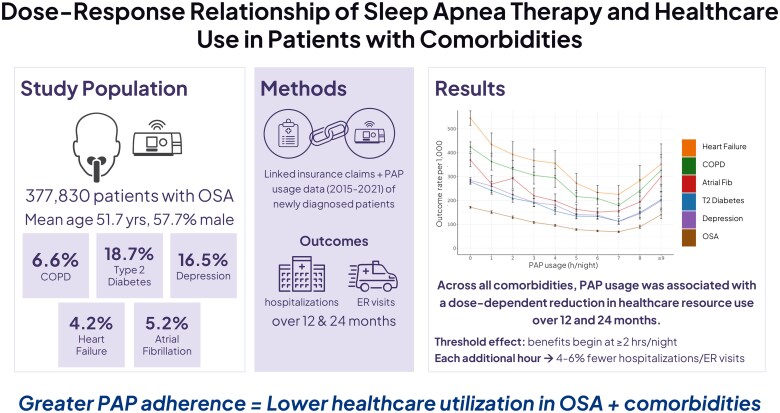

## Introduction

Obstructive sleep apnea (OSA) is a highly prevalent chronic condition around the world [1–3]. OSA is frequently associated with cardiovascular, metabolic, pulmonary, and psychiatric comorbidities [4]. Left untreated, OSA increases the risk of developing several major chronic conditions and is associated with increased all-cause mortality [5–9]. In addition, due to shared risk factors and underlying mechanisms of disease, complex interactive relationships between OSA and a number of conditions have been suggested, including chronic obstructive pulmonary disease (COPD) [10, 11], type 2 diabetes [12], depression [13], heart failure [14], and atrial fibrillation [15]. Moreover, existing literature suggest that OSA treatment may be particularly impactful for these potentially high-risk patients [16–19].

Treatment of OSA with positive airway pressure (PAP) therapy has been shown to improve functional outcomes in randomized clinical trials [20–22]. However, due to variable adherence, the benefits of PAP therapy are sometimes questioned. We have previously demonstrated improvements in healthcare resource utilization (HCRU) associated with adherence to PAP therapy in populations with major comorbid conditions [16–18, 23–25]. Currently, US Centers for Medicare & Medicaid Services (CMS) limits reimbursement based on 4 hours/night of use for 70% of nights within a consecutive 30-day window in the first 90 days of therapy. However, these criteria are relatively arbitrary, leading to discussion about developing new data-driven criteria [26]. We have previously reported a dose–response relationship in a general OSA population with increasing PAP usage associated with reduced hospitalizations and emergency room (ER) visits [27], but this relationship has not yet been assessed in patients with major comorbidities.

Based on this conceptual framework, we sought to test the hypothesis that PAP therapy use would be associated with improvements in HCRU in a dose-dependent manner in patients with OSA and chronic comorbid conditions. We specifically focused on conditions that are highly prevalent and frequently comorbid with OSA, including COPD, type 2 diabetes, depression, heart failure, and atrial fibrillation. In addition, we refreshed the previously conducted analysis [27] in a general OSA population with additional patients available for analyses.

## Methods

### Study design and data sources

This retrospective cohort study was conducted using a linked data set that combined de-identified payer-sourced administrative medical and pharmacy claims data from more than 100 geographically dispersed health plans across the United States (licensed from Inovalon Insights LLC, Bowie MD) and individual objective patient PAP usage data from cloud-connected devices (via AirView; Resmed Corp, San Diego, CA). The databases were linked through a tokenization process, and the resulting database underwent expert determination to ensure patient privacy and compliance with the Health Insurance Portability and Accountability Act. The study design was reviewed by an institutional review board (Advarra, ref. Pro0004005) and deemed exempt from oversight.

### Study participants

This study examined a cohort of patients with newly diagnosed OSA, as well as five subcohorts defined based on the presence of comorbid conditions in the year prior to OSA diagnosis.

*OSA cohort*: The OSA cohort included adults (age ≥ 18 years) who underwent a sleep test, subsequently received an OSA diagnosis within 60 days, and initiated PAP therapy (index date) using an AirSense 10 or AirSense 11 device (Resmed Corporation, San Diego, CA) between January 2015 and May 2021. Only those patients with claims data for at least 1 year before and at least 2 years after index were eligible for inclusion. Patients with evidence of PAP resupply in the year before index, pregnancy [28], end-stage renal disease [29], dialysis use, central sleep apnea, or nocturnal hypoventilation at any time in the study period were excluded.

*Comorbid subcohorts:* From the OSA cohort, subcohorts were created with the following comorbid conditions in addition to OSA: (1) COPD, (2) type 2 diabetes, (3) depression, (4) heart failure, and (5) atrial fibrillation. Subcohorts were identified by the presence of at least two healthcare encounters or at least one hospitalization with the relevant comorbidity diagnosis code in the year prior to index. The type 2 diabetes cohort also excluded patients with evidence of a type 1 diabetes diagnosis. Subcohorts were not mutually exclusive, as patients could have multiple comorbid conditions. Codes used to define the cohorts are shown in Table S1 in the Supplementary material.

### Outcomes and predictors

Within each subcohort, the numbers of all-cause hospitalizations and ER visits were examined within 12 and 24 months after index. The primary predictor of interest was the average hours of PAP use per night (total hours used divided by the number of days) within each time frame. Average PAP usage was analyzed as both a continuous variable and categorized into ten 1-hour increments (i.e. <1, 1 to <2, through to ≥ 9 hours/night).

Common covariates of interest across all cohorts included patient demographics (age at index, payer, gender, and obesity status), comorbid conditions (including coronary artery disease, atrial fibrillation, hypertension, cerebrovascular disease, other arrhythmia, atrial flutter, heart failure, asthma, COPD, pneumonia, psychotic disorders, other mood disorders, depression, anxiety, type 2 diabetes, hyperlipidemia, gastroesophageal reflux disease, and cancer) defined on the basis of diagnoses present in the year before index, and prior year HCRU (all-cause hospitalizations and ER visits). The defining comorbid condition in each subcohort was excluded as a covariate from analyses for that cohort. As a proxy for healthy behaviors, adherence to medications (statins, antidepressants, antihypertensives, and beta blockers only), flu vaccines, and wellness visits were also included in all analyses (see Table S1 in the Supplementary material for codes used). Adherence to medication was defined using a prescription exposure window of 181–360 days before starting PAP therapy. Patients who filled a prescription for a medication of interest were considered to be on that medication. For those with a given medication, adherence was defined as a proportion of days covered of at least 80%.

The following cohort-specific variables were included in the comorbid condition subanalyses: number of hospitalizations and ER visits with COPD as primary diagnosis and number of COPD medication prescriptions (in the COPD subcohort); cardioversion, atrial fibrillation medications and oral anticoagulant adherence, CHA2DS2-VASc score (congestive heart failure, hypertension, age ≥ 75 [doubled], diabetes, stroke [doubled], vascular disease, age 65–74, and sex category [female]), and type of atrial fibrillation (in atrial fibrillation subcohort).

### Statistical analysis

Analyses to assess the effects of increased PAP usage over different time frames were conducted within the OSA and all comorbid subcohorts. Within each time frame, the numbers of all-cause hospitalizations and ER visits were capped at the 99.5 percentile and modeled using negative binomial regression to assess the effects of increased PAP usage within 12 and 24 months. The coefficients of all potential confounders were used to generate a single risk score for each patient that was included as a covariate in subsequent risk-adjusted models. Uncapped ER visits and hospitalizations were annualized, and crude and risk-standardized rates per 1000 people (with 95% confidence intervals [30]) were calculated using the “epitools” package in R [31], and plotted against PAP usage categories.

As demonstrated previously [27, 32], both the minimum threshold to confer a significant benefit and the incremental benefit with increased use were assessed. The minimum threshold was determined by analyzing categorical PAP usage and identifying the first category that differed significantly (*p* < .05) from the reference of < 1 hour per night. Incremental benefit was modeled with PAP usage as a continuous variable. Modeled event rates were reported per 1000 people. The percent reduction in the number of outcomes with each additional hour of PAP use was calculated by exponentiating the model coefficient to derive the incidence rate ratio with 95% confidence interval and subtracting from 1. The absolute reduction in model-predicted events per 1000 people was estimated by applying the percent reduction to the overall event rate predicted by the model. All analyses were conducted using R (version 4.2.2) [33].

## Results

### Demographics and clinical characteristics

There were 377 830 patients included in the OSA cohort and 24 866 with COPD, 70 533 with type 2 diabetes, 62 316 with depression, 16 045 with heart failure, and 19 784 with atrial fibrillation in the subcohorts. The average age was 51.7 years in the OSA cohort (average age range: 50.7–62.0 years). The OSA cohort was majority male (57.7%), as were all subcohorts except for COPD (45.6%) and depression (37.6%). More than half of patients from the heart failure subcohort had severe obesity, and they had the highest comorbidity burden with an average of 6.13 conditions other than heart failure. Baseline demographics and comorbid conditions for all cohorts are described in Table 1.

**Table 1:** Baseline characteristics for the OSA cohort and comorbid condition subcohorts

**Characteristic, no. (%)**	**OSA**	**COPD**	**Type 2 diabetes**	**Depression**	**Heart failure**	**Atrial fibrillation**
	***n* = 377 830**	***n* = 24 866**	***n* = 70 533**	***n* = 62 316**	***n* = 16 045**	***n* = 19 784**
*Demographics*						
Female	159 882 (42.3)	13 521 (54.4)	34 070 (48.3)	38 876 (62.4)	7 386 (46.0)	6 790 (34.3)
Age (years), mean ± SD	51.7 ± 11.9	59.9 ± 10.5	56.6 ± 10.9	50.7 ± 12.6	60.6 ± 11.8	62.0 ± 10.9
Obesity/BMI (kg/m^2^)						
Severe obesity (≥40)	103 315 (27.3)	9 602 (38.6)	30 517 (43.3)	22 401 (36.0)	8 153 (50.8)	6 580 (33.3)
Obesity (≥30–<40)	111 164 (29.4)	7 423 (29.9)	21 398 (30.3)	18 402 (29.5)	4 322 (26.9)	6 311 (31.9)
Overweight (≥25–<30)	25 552 (6.8)	1 638 (6.6)	2 998 (4.3)	3 999 (6.4)	740 (4.6)	1 490 (7.5)
No obesity indicated	137 799 (36.5)	6 203 (25.0)	15 620 (22.2)	17 514 (28.1)	2 830 (17.6)	5 403 (27.3)
Number of conditions^a^, mean ± SD	3.0 ± 2.3	5.3 ± 2.6	4.0 ± 2.3	3.8 ± 2.4	6.1 ± 2.5	4.9 ± 2.6
*Cardiac conditions*						
Coronary artery disease	44 992 (11.9)	8 980 (36.1)	16 880 (23.9)	9 034 (14.5)	9 063 (56.5)	7 717 (39.0)
Atrial fibrillation	22 684 (6.0)	3 485 (14.0)	6 650 (9.4)	3 422 (5.5)	5 579 (34.8)	19 784 (100.00)
Pulmonary hypertension	7 503 (2.0)	2 412 (9.7)	2 805 (4.0)	1 592 (2.6)	3 192 (19.9)	1 920 (9.7)
Hypertension	217 753 (57.6)	20 538 (82.6)	61 171 (86.7)	38 274 (61.4)	15 112 (94.2)	16 785 (84.8)
Cerebrovascular disease	18 733 (5.0)	3 302 (13.3)	6 624 (9.4)	4 694 (7.5)	2 661 (16.6)	2 803 (14.2)
Other arrhythmia	29 480 (7.8)	3 947 (15.9)	7 587 (10.8)	5 634 (9.0)	5 004 (31.2)	7 782 (39.3)
Atrial flutter	4 712 (1.3)	758 (3.1)	1 391 (2.0)	675 (1.1)	1 466 (9.1)	3 823 (19.3)
Heart failure	21 848 (5.8)	6 589 (26.5)	9 584 (13.6)	4 960 (8.0)	16 045 (100.0)	6 211 (31.4)
*Respiratory conditions*						
Asthma	59 154 (15.7)	9 819 (39.5)	14 674 (20.8)	14 531 (23.3)	4 436 (27.7)	3 335 (16.9)
COPD	34 012 (9.0)	24 866 (100.0)	11 961 (17.0)	9 004 (14.5)	6 274 (39.1)	3 915 (19.8)
Pneumonia	15 817 (4.2)	4 701 (18.9)	5 177 (7.3)	4 136 (6.6)	3 268 (20.4)	2 069 (10.5)
*Affective conditions*						
Psychotic disorders	16 190 (4.3)	2 749 (11.1)	4 288 (6.1)	7 615 (12.2)	1 046 (6.5)	516 (2.6)
Other mood disorders	34 245 (9.1)	2 695 (10.8)	6 400 (9.1)	14 024 (22.5)	1 375 (8.6)	1 233 (6.2)
Depression	89 406 (23.7)	9 291 (37.4)	20 155 (28.6)	62 316 (100.0)	4 931 (30.7)	4 018 (20.3)
Anxiety	90 656 (24.0)	8 752 (35.2)	17 518 (24.8)	37 023 (59.4)	4 536 (28.3)	4 291 (21.7)
*Other conditions*						
Type 2 diabetes	86 864 (23.0)	10 953 (44.1)	70 533 (100.0)	17 654 (28.3)	8 645 (53.9)	6 796 (34.4)
Hyperlipidemia	192 388 (50.9)	17 935 (72.1)	56 396 (80.0)	34 379 (55.2)	12 500 (77.9)	14 337 (72.5)
GERD	103 638 (27.4)	11 637 (46.8)	24 492 (34.7)	25 042 (40.2)	6 385 (39.8)	6 531 (33.0)
Cancer	24 594 (6.5)	2 789 (11.2)	5 869 (8.3)	4 483 (7.2)	1 780 (11.1)	2 406 (12.2)
*Prior year HCRU*						
Had ≥ 1 hospitalization	32 000 (8.7)	7 587 (30.5)	11 443 (16.2)	10 436 (16.8)	7 154 (44.6)	5 932 (30.0)
Had ≥ 1 emergency room visit	109 132 (28.9)	13 362 (53.7)	27 705 (39.3)	26 741 (42.9)	9 052 (56.4)	2 914 (14.7)
*Healthy behavior proxies*						
Flu vaccinations	105 108 (27.8)	8 982 (36.1)	24 873 (35.3)	20 752 (33.3)	5 607 (35.0)	6 943 (35.1)
Wellness visits	152 430 (40.3)	7 729 (31.1)	24 427 (34.6)	24 490 (39.3)	4 649 (29.0)	7 055 (35.7)
Adherence to statins						
Adherent	60 134 (16.0)	6 793 (27.3)	22 684 (32.2)	10 985 (17.6)	4 859 (30.3)	5 495 (27.8)
Not adherent	32 144 (8.5)	3 514 (14.1)	11 797 (16.7)	6 110 (9.8)	2 379 (14.8)	2 136 (10.8)
Not present	285 372 (75.5)	14 559 (58.6)	36 052 (51.1)	45 221 (72.6)	8 807 (54.9)	12 153 (61.4)
Adherence to antidepressants						
Adherent	66 416 (17.6)	6 228 (25.1)	14 549 (20.6)	30 025 (48.2)	2 937 (18.3)	2 646 (13.4)
Not adherent	32 450 (8.6)	2 936 (11.8)	6 636 (9.4)	11 589 (18.6)	1 519 (9.5)	1 261 (6.4)
Not present	278 964 (73.8)	15 702 (63.2)	49 348 (67.0)	20 702 (33.2)	11 589 (72.2)	15 877 (80.3)
Adherence to antihypertensives						
Adherent	110 406 (29.2)	11 550 (46.5)	36 418 (51.6)	19 738 (31.7)	9 373 (58.4)	9 565 (48.4)
Not adherent	32 866 (8.7)	2 924 (11.8)	8 759 (12.4)	6 313 (10.1)	1 886 (11.8)	1 856 (9.4)
Not present	234 558 (62.1)	10 392 (41.8)	25 356 (36.0)	36 265 (58.2)	4 786 (29.8)	8 363 (42.3)
Adherence to beta blockers						
Adherent	39 663 (10.5)	4 913 (19.8)	13 615 (19.3)	7 472 (12.0)	5 560 (34.7)	6 671 (33.7)
Not adherent	19 262 (5.1)	2 495 (10.0)	5 797 (8.2)	4 329 (7.0)	2 574 (16.0)	2 527 (12.8)
Not present	318 905 (84.4)	17 458 (70.2)	51 121 (72.5)	50 515 (81.1)	7 911 (49.3)	10 586 (53.5)

a Cohort defining comorbidity is excluded from number of conditions for each cohort

COPD, chronic obstructive pulmonary disease; GERD, gastroesophageal reflux disease; HCRU, healthcare resource utilization; OSA, obstructive sleep apnea, SD, standard deviation.

### PAP therapy usage

On average, patients with OSA used PAP therapy for 3.8 hours per night in the first 12 months after index. The subcohorts generally had lower PAP therapy use than the OSA cohort (ranging from 3.2 to 3.5 hours per night), with the exception of patients with atrial fibrillation, who used PAP therapy for an average of 4.1 hours per night. Across all cohorts, PAP therapy usage declined in months 12–24. PAP therapy use in the first 24 months is described in Table 2.

**Table 2:** PAP usage in months 1–12 and 12–24 for the OSA cohort and comorbid condition subcohorts

**PAP usage, mean ± SD**	**OSA** ***n* = 377 830**	**COPD** ***n* = 24 866**	**Type 2 diabetes** ***n* = 70 533**	**Depression** ***n* = 62 316**	**Heart failure** ***n* = 16 045**	**Atrial fibrillation** ***n* = 19 784**
*First 12 months*						
Days per week	4.3 ± 2.6	3.6 ± 2.7	4.0 ± 2.6	3.8 ± 2.6	3.8 ± 2.7	4.5 ± 2.6
Hours per night	3.8 ± 2.8	3.2 ± 2.9	3.5 ± 2.9	3.4 ± 2.9	3.4 ± 2.9	4.1 ± 2.8
Hours per use night	5.5 ± 2.0	5.1 ± 2.3	5.3 ± 2.1	5.4 ± 2.1	5.2 ± 2.3	5.5 ± 2.1
CMS compliance^a^, no. (%)	259 439 (68.7)	14 245 (57.3)	45 308 (64.2)	39 107 (62.8)	9 630 (60.0)	14 076 (71.2)
*Months 12–24*						
Days per week	3.4 ± 3.0	2.7 ± 3.0	3.0 ± 3.0	2.8 ± 3.0	3.0 ± 3.0	3.8 ± 3.0
Hours per night	3.3 ± 3.2	2.7 ± 3.3	2.9 ± 3.2	2.7 ± 3.2	2.9 ± 3.3	3.6 ± 3.3
Hours per use night	6.0 ± 1.9	5.9 ± 2.4	5.9 ± 2.1	6.0 ± 2.2	5.9 ± 2.4	6.2 ± 2.0

a CMS compliance is defined as using for at least 4 hours per night on at least 70% of nights in a consecutive 30-day window in the first 90 days of therapy.

CMS, Centers for Medicare & Medicaid Services; COPD, chronic obstructive pulmonary disease; OSA, obstructive sleep apnea; PAP, positive airway pressure; SD, standard deviation.

### Risk factors for hospitalizations and ER visits

Generally, increased HCRU in the year before index was a major significant predictor of higher hospitalizations and ER visits after index. Other common significant predictors of hospitalizations and ER visits across time points and cohorts included Medicaid or dual Medicaid/Medicare Advantage insurance and a higher comorbidity burden.

### Relationship between PAP usage and HCRU: minimum usage threshold to confer benefit and incremental benefit

**Figure 1:**
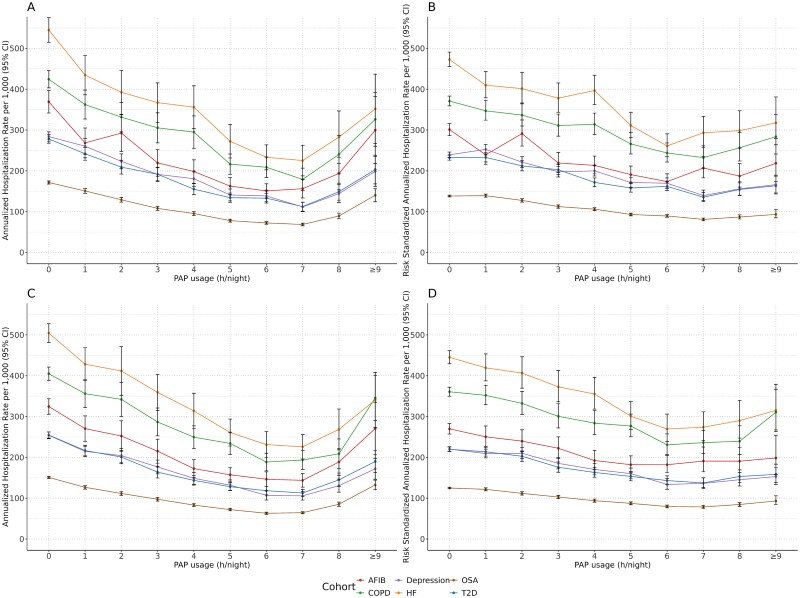
Annualized rate per 1000 of (A) 12-month all-cause hospitalizations (crude); (B) 12-month all-cause hospitalizations (risk adjusted); (C) 24-month all-cause hospitalizations (crude); and (D) 24-month all-cause hospitalizations (risk adjusted). 95% CI = 95% confidence interval; AFIB = atrial fibrillation; COPD = chronic obstructive pulmonary disease; HF = heart failure; OSA = obstructive sleep apnea; PAP = positive airway pressure; T2D = type 2 diabetes.

**Figure 2:**
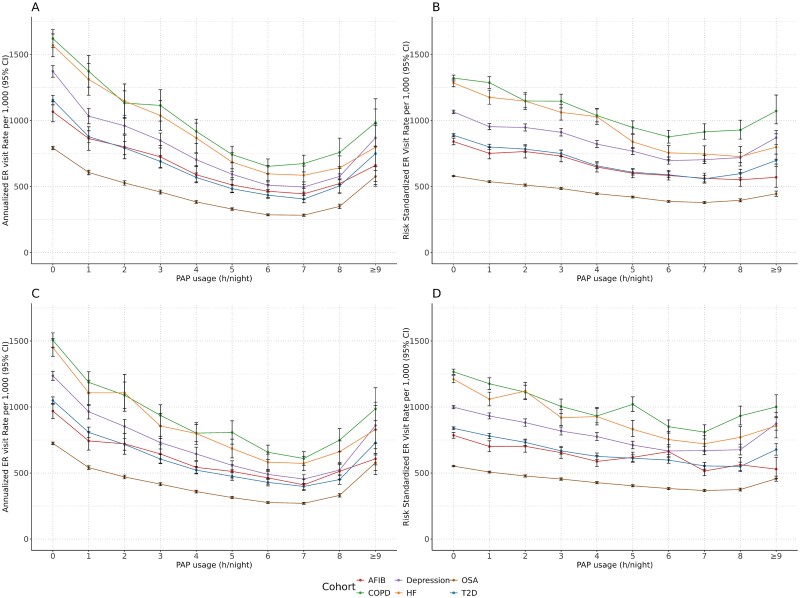
Annualized rate per 1000 of (A) 12-month ER visits rate (crude); (B) 12-month ER visits rate (risk adjusted); (C) 24-month ER visits rate (crude); and (D) 24-month ER visits rate (risk adjusted). 95% CI = 95% confidence interval; AFIB = atrial fibrillation; COPD = chronic obstructive pulmonary disease; HF = heart failure; OSA = obstructive sleep apnea; PAP = positive airway pressure; T2D = type 2 diabetes.

In the 12 and 24 months after index, increased PAP usage was associated with decreased HCRU. Across all cohorts, crude event rates increased slightly when PAP usage was at least 8 hours per night but remained below those with less than 1 hour of PAP usage per night (Figures 1A and C and 2A and C). However, this increase was moderated after adjusting for the distribution of risk scores (derived from all included covariates) within each cohort (Figures 1B and D and 2B and D).

Capped values (99.5 percentile) for each cohort are shown in Table S2 in the Supplementary material. In the OSA cohort, capped values at 12 and 24 months were 3 and 5 for hospitalizations, and 8 and 14 for ER visits, respectively. Caps for subcohorts ranged from 4 to 5 and 6 to 9 for hospitalizations, and from 9 to 15 and 16 to 26 for ER visits at 12 and 24 months, respectively.

After risk adjustment, patients in the OSA cohort attained significant benefit starting at 2 to less than 3 hours per night for hospitalizations and 1 to less than 2 hours of usage per night for ER visits across both time points. The minimum threshold to derive a significant benefit (in hospitalizations or ER visits) in the subcohorts was at most 3 to less than 4 with COPD, 2 to less than 3 with type 2 diabetes, 3 to less than 4 with depression, 2 to less than 3 with heart failure, and 3 to less than 4 with atrial fibrillation, hours per night across both time points (Table 3).

**Table 3:** Risk-adjusted minimum positive airway pressure usage threshold and incremental benefit for the OSA and comorbid condition subcohorts

	**Hospitalizations**		**Emergency room visits**	
	**12 months**	**24 months**	**12 months**	**24 months**
*Predicted event rate per 1 000 patients*				
OSA	111	208	471	917
COPD	319	640	1 154	2 222
Type 2 diabetes	193	368	728	1 412
Depression	203	377	889	1 702
Heart failure	382	757	1 046	2 022
Atrial fibrillation	226	437	658	1 266
*Minimum usage for benefit* *^a^* *, h/night*				
OSA	2	2	1	1
COPD	3	2	2	2
Type 2 diabetes	2	2	1	1
Depression	3	2	1	1
Heart failure	1	2	2	1
Atrial fibrillation	1	2	3	1
*Percent reduction per additional hour of PAP usage* *^b^* *(95% CI)*				
OSA	6.8 (6.4-7.1)	5.8 (5.5-6.1)	5.4 (5.2-5.7)	4.9 (4.8-5.1)
COPD	5.2 (4.3-6.2)	4.7 (4.0-5.5)	4.6 (4.0-5.2)	4.1 (3.6-4.7)
Type 2 diabetes	6.1 (5.4-6.8)	5.5 (4.9-6.0)	5.6 (5.1-6.0)	5.1 (4.8-5.5)
Depression	6.1 (5.4-6.8)	5.9 (5.3-6.5)	5.1 (4.6-5.6)	4.6 (4.2-5.0)
Heart failure	6.2 (5.1-7.3)	5.8 (4.9-6.7)	6.2 (5.4-7.0)	5.4 (4.7-6.0)
Atrial fibrillation	5.8 (4.6-7.0)	5.1 (4.2-6.1)	5.2 (4.3-6.0)	4.7 (4.1-5.4)
*Absolute reduction in events per additional hour of PAP usage, per 1 000 patients* *^c^* *(95% CI)*				
OSA	7.5 (7.1-7.9)	12.1 (11.5-12.8)	25.6 (24.5-26.7)	45.3 (43.6-46.9)
COPD	16.8 (13.7-19.8)	30.3 (25.3-35.3)	53.3 (46.1-60.5)	91.9 (80.2-103.5)
Type 2 diabetes	11.8 (10.5-13.2)	20.1 (18.0-22.2)	40.6 (37.4-43.9)	72.7 (67.6-77.7)
Depression	12.4 (10.8-13.9)	22.1 (19.8-24.5)	45.4 (41.3-49.4)	78.8 (72.3-85.3)
Heart failure	23.7 (19.5-27.9)	43.7 (36.9-50.4)	65.0 (56.7-73.3)	108.6 (95.1-121.9)
Atrial fibrillation	13.1 (10.3-15.8)	22.5 (18.2-26.7)	34.0 (28.5-39.4)	60.1 (51.8-68.4)

a Divided into usage categories (i.e. 0 = 0 to <1, 1 = 1 to <2, 2 = 2 to <3, through to ≥ 9).

b Calculated as 1 − IRR.

c Based on the percent reduction and lower/upper confidence limits applied to the predicted event rate.

CI, confidence interval; COPD, chronic obstructive pulmonary disease; ER, emergency room; IRR, incidence rate ratio; OSA, obstructive sleep apnea; PAP, positive airway pressure.

In every cohort, increasing PAP usage was associated with a statistically significant decline in all-cause hospitalizations and ER visits at both 12 and 24 months (all analyses *p* < .0001). In the OSA cohort, for each additional hour of PAP usage per night, there was a 4.9%–6.8% reduction in hospitalization and ER visit rates (4.1%–5.2% in COPD, 5.1%–6.1% in type 2 diabetes, 4.6%–6.1% in depression, 5.4%–6.2% in heart failure, and 4.7%–5.8% in atrial fibrillation reduction in hospitalization and ER visit rates with each additional hour per night of PAP usage in the subcohorts). The corresponding absolute reductions (per 1000 patients) with each additional hour of PAP usage are listed in Table 3.

## Discussion

This real-world study adds to the literature in a number of important ways. First, with an expanded and updated cohort, we reinforce the robustness of data showing that increased PAP usage was associated with decreased HCRU over the first 2 years of treatment in a dose-dependent manner [27, 34]. In every comorbid subcohort, increasing PAP usage was associated with a statistically and clinically significant decline in all-cause hospitalizations and ER visits over both 12 and 24 months. Second, after risk adjustment, the minimum threshold to derive a significant benefit in the comorbid subcohorts was at most 3 to less than 4 hours per night of PAP usage across time points. Current CMS guidelines require at least 4 hours per night for reimbursement, potentially leaving patients without treatment coverage that would benefit from therapy. Third, we observed that with each additional hour of PAP usage, there was a 4.1%–6.2% reduction per hour in hospitalization and ER visit rates across the comorbid subcohorts. Of note, similar benefits (dose response, incremental, and minimum usage) are seen from PAP therapy across these five subcohorts, despite variations in event rates, which highlights the broad applicability of PAP therapy. Fourth, we presented a comprehensive list of conditions within each subcohort, revealing high disease burden across the board. Patients with heart failure or COPD had an average of over five comorbid conditions and high baseline HCRU. In addition, PAP usage varied across subcohorts, with patients with atrial fibrillation demonstrating the highest usage rates and those with COPD the lowest. These findings highlight the heterogeneity of profiles of patient with OSA and may inform future efforts to tailor engagement and management strategies by comorbidity profile.

Although the clinical benefits of PAP therapy are well established, there has been some skepticism regarding improvement in cardiovascular outcomes in these patients. Several randomized trials have shown no major benefit from PAP therapy with regards to cardiovascular outcomes in OSA [35–37]. The reason for this finding is unclear but may be related to patient selection and/or adherence to PAP therapy [22, 38]. Ongoing efforts to determine the optimal patients to enroll in subsequent clinical trials will need to be guided by rigorous data. Our observation that patients with major comorbidities have marked improvements in HCRU with PAP therapy as a function of hours of PAP usage may help to inform these decisions. Given that we observed marked improvements in all of the cohorts of patients with OSA and major comorbidities, one could argue that such patients may be good candidates for subsequent clinical trials. On the other hand, some have argued that treating patients with OSA without comorbidities may be the most impactful if major complications can be prevented before major disease becomes established [39]. Ultimately, large scale clinical trials may be required to inform this debate [40]. However, our data strongly suggest that patients with OSA and major comorbid conditions are likely to respond well to PAP therapy, even when usage levels are below CMS thresholds, which supports ongoing advocacy efforts to change the PAP coverage requirements [26].

Our study has several strengths. We were able to analyze a large number of real-world patients using administrative claims linked to objective PAP usage data to evaluate important health outcomes. The comprehensive information captured in the closed claims dataset enabled adjustment for a wide array of major confounders. However, we acknowledge a number of limitations. Although we performed rigorous analyses, we recognize that, as with all observational studies, residual confounding may still be present. Of note, severity of comorbid conditions, which may affect both PAP adherence and HCRU, is not available in the claims data. Another potential source is a healthy user effect, as adherence to PAP therapy may be associated with other healthy behaviors [41], including adherence to medications, diet, and exercise [42]. Using available data, we adjusted for multiple healthy behaviors including vaccinations, wellness visits, and medication adherence. It is important to note that this analysis was also adjusted for HCRU in the year prior to starting PAP to help mitigate possible healthy user confounding, as prior research has shown that PAP adherence is associated with health-seeking behaviors and lower HCRU prior to PAP initiation [43]. Thus, it is unlikely that our findings are driven primarily by a healthy user effect, but subsequent randomized trials to further examine this question remain important. Furthermore, given the observational nature of the study, we are unable to delineate the underlying mechanisms of why excessive PAP usage may be associated with various outcomes. Our data are consistent with existing literature regarding sleep duration and PAP usage [44–46], but we advocate strongly for further research in this area. It should also be noted that we examined a predefined set of comorbidities in isolation, rather than an exhaustive list of conditions commonly associated with OSA. As such, we are unable to draw any major conclusions about patients with some other specific comorbidity or multiple comorbidities (e.g. coronary artery disease, COPD plus heart failure) from this analysis, although this remains a question of interest. In addition, because the inclusion in the study required continuous insurance enrollment for the full 24 months after index, individuals who died during that time were excluded, which may introduce a survivorship bias and limit the generalizability of findings for patients with more severe disease or higher mortality risk. Lastly, there are a number of important variables that are not available in administrative claims data, including patient-reported outcomes [47–49]. Using administrative claims data to define conditions may also introduce diagnostic misclassification, particularly for clinically overlapping conditions such as asthma and COPD. Despite these limitations, we believe that our new findings add valuable knowledge to the existing literature in this area and may be clinically directive until additional data are available to provide further guidance.

## Conclusions

This real-world analysis of patients with OSA and major comorbid conditions showed a dose-dependent relationship between PAP usage and HCRU. We saw a 4%–6% reduction in events per incremental hour of PAP usage and reduced HCRU starting below 4 hours per night over 12 and 24 months. These results should be used to inform data-driven reimbursement guidelines for PAP therapy and support the treatment of OSA in patients with other major comorbid conditions.

## Supplementary Material

Clean_Dose-Response_relationship_between_OSA_PAP_therapy_and_HCRU_zsaf333

## Data sharing statement

The data are not publicly available due to privacy restrictions.
