# Supplementary material for: Dose–response relationship of sleep apnea therapy and healthcare use in patients with comorbidities
Source: Sleep. 2025 Oct 27;49(3):zsaf333. doi: 10.1093/sleep/zsaf333 (PMC13017776; doi:10.1093/sleep/zsaf333)
Supplement: Clean_Dose-Response_relationship_between_OSA_PAP_therapy_and_HCRU_zsaf333 [file clean_dose-response_relationship_between_osa_pap_therapy_and_hcru_zsaf333.docx]

# Supplemental Materials

## Dose-Response Relationship of Sleep Apnea Therapy and Healthcare Use in Patients with Comorbidities

Atul Malhotra^1^, Suyog More^2^, Naomi Alpert^3^, Jean-Louis Pépin^4^, Kate V Cole^3^, Caleb Woodford^2^, Adam V Benjafield^5^, Peter A Cistulli^6^, and Kimberly L Sterling^3^ on behalf of the medXcloud Group

1 University of California San Diego, La Jolla CA, USA

2 Resmed Science Centre, Halifax NS, Canada

3 Resmed Science Center, San Diego CA, USA

4 HP2 Laboratory (Hypoxia: Pathophysiology), Grenoble Alpes University, Grenoble, France

5 Resmed Science Center, Sydney, Australia

6 Charles Perkins Centre and Faculty of Medicine and Health, University of Sydney, Sydney, Australia

**Correspondence and requests for reprints should be addressed to**: Atul Malhotra, M.D., University of California, San Diego, 9300 Campus Point Drive, #7381, La Jolla, CA 92037. E-mail: [amalhotra@ucsd.edu](mailto:amalhotra@ucsd.edu)

## Table S1. Codes used to identify conditions and procedures

| **Comorbidity/procedure** | **Code** |
| --- | --- |
| OSA | *ICD-10-CM:* G47.33 |
| COPD | *ICD-10-CM:* J41-J44 |
| Type 2 diabetes | *ICD-10-CM: E11* |
| Heart Failure | *ICD-10-CM:* I50, I110, I130, I97130, I97131, I0981 |
| Atrial Fibrillation | *ICD-10-CM:* I480, I481, I482, I4891 |
| Depression | *ICD-10-CM:* F32, F33, F34, F39 |
| Type 1 Diabetes | *ICD-10-CM:* E10 |
| Sleep Test | *HCPCS*: 95808, 95810, 95811, G0398–G0400 |
| Flu Vaccines | *CPT:* 90630, 90653- 90658, 90660-90662, 90664, 90666-90668, 90672-90674, 90682, 90685-90689, 90756 |
| Wellness Visits | *CPT:* G0402, G0438, G0439, G0468, 99385-99387, 99395-99397  ICD-9-CM: V70.0 |

COPD = chronic obstructive pulmonary disease; ICD-10-CM = the International Classification of Diseases, Tenth Revision, Clinical Modification; OSA = obstructive sleep apnea

## Table S2. Capped values at 99.5 percentile

|  | **Hospitalizations** | | **Emergency Room Visits** | |
| --- | --- | --- | --- | --- |
| **Capped values** | **12 Month** | **24 Month** | **12 Month** | **24 Month** |
| OSA | 3 | 5 | 8 | 14 |
| COPD | 5 | 9 | 15 | 26 |
| Type 2 Diabetes | 4 | 6 | 11 | 19 |
| Depression | 4 | 7 | 14 | 24 |
| Heart Failure | 5 | 9 | 13 | 24 |
| Atrial Fibrillation | 4 | 7 | 9 | 16 |

COPD = chronic obstructive pulmonary disease; OSA = obstructive sleep apnea
